# Professional roles and activities of simulated patient educators. Development of a multidimensional competency framework

**DOI:** 10.3205/zma001877

**Published:** 2026-09-15

**Authors:** Daniel Bauer, Tanja Hitzblech, Peter Eberz, Angelika H. Fritz, Veronika Kühn, Tim Peters, Swetlana Philipp, Renate Strohmer, Beate G. Brem

**Affiliations:** 1 University of Bern, Institute for Medical Education, Bern, Switzerland; 2 Charité – University Medical Center Berlin, corporate member of Freie Universität Berlin and Humboldt-Universität zu Berlin, Berlin, Germany; 3 University of Duisburg-Essen, Faculty of Medicine, Essen, Germany; 4 University of Ulm, Medical Faculty, Ulm, Germany; 5 Universität Bielefeld, Med. Fakultät OWL, Department of Studies and Teaching, Department of General Practice and Family Medicine, Bielefeld, Germany; 6 Friedrich-Schiller University, Jena University Hospital, Institute for Psychosocial Medicine, Psychotherapy and Psychooncology, Jena, Germany; 7 Heidelberg University, Medical Faculty Mannheim, Division of Studies and Teaching Development, SkillsLab TheSiMa, Simulation Person Program, Mannheim, Germany

**Keywords:** simulated patient educator, simulated participants, simulation-based rducation, competency framework, health professions education, professionalization

## Abstract

Standardized Participant (SP) Educators (SPEs) play a central role in simulation-based education, yet their professional profile and competencies have so far remained insufficiently defined. Against this background, the aim of this project was to develop a competency-based framework for SPEs that reflects their professional roles, core activities, and required competencies within health professions education.

An expert group from Germany and Switzerland, representing diverse academic and professional backgrounds, collaboratively developed the framework through an inductive, participatory, and consensus-driven process. Established competency frameworks from health professions education, including CanMEDS and the German NKLM, served as conceptual references. The development process included iterative feedback loops and a final cross-comparison with published international frameworks to ensure completeness and contextual validity.

The resulting framework defines eight professional roles and 39 professional activities, subdivided into 132 tasks and associated competencies. It provides a comprehensive, practice-oriented, and formative tool that captures the complexity and scope of SPEs’ work. By mapping roles, activities, and competencies, it offers orientation for program development, team structuring, and capacity building, while highlighting the need for professional recognition of SPEs as qualified academic staff.

While primarily contextualized for German-speaking countries, the framework may also serve as a reference for SP programs internationally and as a foundation for ongoing discourse and future adaptation.

## Abstract (de)

SP-Expert:innen (SPE) spielen eine zentrale Rolle in der simulationsbasierten Lehre. Ihr berufliches Profil sowie die hierfür erforderlichen Kompetenzen sind bislang jedoch nur unzureichend definiert. Vor diesem Hintergrund war das Ziel dieses Projekts die Entwicklung eines kompetenzbasierten Rahmenwerks für SPE, der ihre professionellen Rollen, zentralen Tätigkeiten und erforderlichen Kompetenzen in der Ausbildung der Gesundheitsberufe abbildet.

Eine Expert:innengruppe aus Deutschland und der Schweiz mit unterschiedlichen akademischen und beruflichen Hintergründen entwickelte den Kompetenzrahmen gemeinsam in einem induktiven, partizipativen und konsensbasierten Prozess. Etablierte Kompetenzrahmen der Ausbildung der Gesundheitsberufe, darunter CanMEDS und der deutsche Nationale Kompetenzbasierte Lernzielkatalog Medizin (NKLM), dienten als konzeptionelle Referenzen. Der Entwicklungsprozess umfasste iterative Feedbackschleifen sowie einen abschliessenden Abgleich mit publizierten internationalen Kompetenzrahmenwerken, um Vollständigkeit und kontextuelle Validität sicherzustellen.

Der resultierende Kompetenzrahmen definiert acht professionelle Rollen und 39 professionelle Tätigkeiten, die in 132 Aufgaben sowie zugehörige Kompetenzen untergliedert sind. Er stellt ein umfassendes, praxisorientiertes und formatives Instrument dar, das die Komplexität und den Umfang der Arbeit von SPE darstellt. Durch die Abbildung von Rollen, Tätigkeiten und Kompetenzen bietet er Orientierung für die Programmentwicklung, die Strukturierung von Teams und den Kompetenzaufbau und verdeutlicht zugleich die Notwendigkeit der professionellen Anerkennung von SPE als qualifiziertem akademischem Personal.

Obwohl der Kompetenzrahmen primär für den deutschsprachigen Raum kontextualisiert wurde, kann er auch für internationale SP-Programme als Referenz dienen und eine Grundlage für den weiteren Diskurs sowie zukünftige Anpassungen bilden.

## 1. Background

There is a growing body of literature on employing simulated participants (SPs) in teaching and assessment, and on the value and effectiveness of this method [1], [2], [3]. However, the professional scope of SP educators (SPEs), those who design, deliver, and oversee SP-based learning experiences remains underexplored. Almost no attention has been paid to the specific tasks SPEs perform or to the competencies required to fulfil this role effectively. 

SPEs can be understood as third space professionals, operating at the intersection of academic, clinical, and administrative domains [4], [5]. Their work extends beyond traditional teaching or management roles, as they integrate educational theory, simulation pedagogy, and organizational strategy to create authentic learning experiences. SPEs collaborate closely with faculty, clinicians, and administrative staff, translating curricular goals into practical simulation activities while ensuring quality of teaching, assessment alignment, and learner support. A clearly defined competency framework which ensures role clarity, informs training, and supports professional development is crucial for this hybrid position.

One of the few comprehensive studies on SPEs is that of Kim et al. (2012) [6], which, based on focus group interviews, identifies ten duties, subdivided into 25 tasks and 76 task elements for South Korean SPEs. A particular strength of this work lies in the involvement of multiple medical schools as well as the South Korean medical licensing examination institute.

In 2017, Lewis et al. published the Standards of Best Practice (SOBP) of the Association of Standardized Patient Educators (ASPE), defining five domains, 19 principles, and 97 practices for high-quality SP work, along with five core values. These standards also introduce an inclusive definition of SPEs as professionals who “work to develop expertise in SP methodology and are responsible for training and/or administering SP-based simulation” [7]. While developed through a rigorous process and with the involvement of leading figures in the SP community, the SOBP do not appear to have drawn on Kim et al.’s earlier work.

Further highlighting the influence of cultural context, Brem et al. (2023) conducted an international interview study across SP programs on all continents. They identified both universal elements and culture-specific adaptations in the application of the ASPE SOBP [8]. 

Other publications and methodological guides on SP programs offer only limited insights into the work of SPEs, often providing merely indirect or implicit references [9], [10]. Similarly, the trinational DACH Association for Medical Education’s (DACH is a commonly used acronym referring to Germany (D), Austria (A), and Switzerland (CH)) certification process for SP programs, though closely aligned with the ASPE SOBP, does not elaborate on the specific role or contributions of SPEs [11]. While some conclusions about SPE responsibilities may be inferred from literature on SP performance or feedback [12], [13], these insights remain scattered and unstructured.

In 2020, a comprehensive textbook on SP methodology among else revisited the question: “Who are Standardized Patient Educators and what do they do?” [14]. Yet, the response primarily underscores the heterogeneous professional backgrounds of SPEs. Concerning their specific tasks, the authors mainly reference the work of Pritchard et al. (2017), who interviewed experienced SPEs to explore how they prepare and support SPs [15]. Their framework identifies three pillars and four overarching themes, comprising eight subthemes and 27 components, characterizing well-structured SP programs. Elcin (2020) further contributes to the discussion by offering definitions and descriptions of SPEs beyond the North American context but also notes: “Despite the importance of their roles in SP methodology, SPEs around the world have remained understudied with the lack of a clearly defined job description, and lack of professional development, and career track.” [16]. 

This gap has concrete implications. A clearly defined job description could guide individual and team-based capacity building, highlight the academic nature of much SPE work, even when SPE often time come from outside academia, and justify institutional investment [17]. Indeed, SPEs do far more than train SPs and manage logistics; they contribute substantially to pedagogical quality and program development.

Notably, none of the existing sources adopt a competency-based approach, although such an approach is particularly well-suited for the field of SPEs. Given the wide variation in SP programs and the diverse professional backgrounds of SPEs (e.g., academic vs. non-academic; backgrounds in theatre, education, or health professions), it is precisely the competencies required for their professional activities and the roles they assume in everyday practice that define the core of their work.

Against this background, we aimed at developing a competency-based framework for SPEs to fill the gap as articulated [16], [18], one that is firmly grounded in practice, reflects key professional activities, and articulates the professional roles that define this unique and evolving field.

## 2. Methods

The task was approached by establishing an expert group to systematically collect, sort, and validate data on the work of SPEs and build a competency-based framework for the profession. This process was guided by established frameworks from health professions education and built on the group’s experiences and expertise as well as literature on SPE work.

### 2.1. Team composition and task specification

To address the task, the standing committee on simulated participants of the DACH Association for Medical Education was consulted. After presenting the idea of creating a competency-based framework for SPEs, the authors formally established a working group in 2021, with a mandate to comprehensively define the roles, tasks, and competencies of SPEs and develop a widely accepted and practically relevant consensus.

The group was intentionally diverse, with German and Swiss members of varied levels of experience and backgrounds, both academic and non-academic, across health professions, pedagogy, psychology, and drama education. Several members had scholarly experience in SP methodology or had worked in developing SPE capacity (in this context, capacity development [19] refers to systematically strengthening the competencies, structures, and strategies needed to support SPEs in performing their complex professional roles and advancing sustainable SP programs). The aim was to reflect the broad diversity of SP programs and the range of professionals performing the SPE role.

Initial discussions clarified that the framework should encompass the full range of SPE work, without suggesting that all tasks and competencies must reside within a single individual or team. This acknowledges the diversity in SP programs based on their maturity, scope and available resources.

Additionally, it was agreed that the framework would focus on SPE activities within health professions education, and explicitely include their roles within the “third space” [4], [5], a fluid intersection of academic and professional domains. While activities outside the (presumed) typical SPE role were excluded from the framework, it was recognized that in practice, boundaries may not always be clear, and many SPEs hold other roles, e.g., as lecturers and teachers, simulation technicians or course administrators.

The framework was developed through an iterative, consensual approach over multiple steps, revisiting certain stages, such as literature reviews, several times throughout the process. The results were continuously reviewed, revised, and refined. In detail, the profiles gradually emerged over the course of the working group sessions in the following steps. 

### 2.2. Literature review and brainstorming

In an initial brainstorming session, roles and tasks that SPEs face in their daily work were identified. Working group members contributed based on their site-specific or individual perspectives and academic routines, as well as existing literature, even though, as mentioned above, this literature was found to be quite limited. 

The brainstorming session also led to an agreement on a common process for the next steps. The process was characterized by a structured form of interaction in which individual contributions were systematically collected, made transparent and integrated.

### 2.3. Defining the framework’s dimensions

Due to the very limited results from the literature review, the working group initially oriented itself towards already established competency-based outcome frameworks for health professionals, specifically the Canadian CanMEDS framework [20]. CanMEDS maps physicians' professional work predominantly through three concepts:

1. the professional’s roles: the broad functions or identities a professional assumes in their work (e.g., communicator, collaborator, leader), capturing the why and in what capacity they act,
2. their professional activities: key tasks that professionals perform in practice, which are observable, delegable, and require integration of multiple competencies, and
3. the corresponding competencies necessary to fulfill these.

After the authors organized the collected and sorted material according to these concepts, and it seemed to provide a suitable representation of the work of SPEs, the group agreed to proceed with this approach and build their framework around the three dimensions of SPEs’ professional roles, professional activities and necessary competencies accordingly. It was beneficial that CanMEDS has equivalents in both the German (National Competency-Based Catalogue of Learning Objectives for Medicine; NKLM) [21] and the Swiss (Principal Relevant Objectives and Framework for Integrative Learning and Education in Switzerland; PROFILES) [22], [23] outcome frameworks for medical education. Alignment of the SPE framework with these familiar frameworks was expected to increase the acceptance.

Sub-working groups were established, one to address the different roles of SPE and the other to compile the professional activities of SPEs, from which the associated competencies were subsequently derived.

### 2.4. Defining the roles and professional activities of SPE

One of the two subgroups collaboratively drafted an initial version of the various roles assumed by SPEs, drawing heavily on the CANMEDs-based roles described in the German NKLM [21]. The role of “medical expert” was adapted and renamed “SP educator”; the roles of scholar, communicator, collaborator, health advocate, professional, and manager were conceptually adapted. Additionally, the role of “visionary”, which is included in the updated version of the NKLM [https://www.nklm.de/] but not part of CanMEDS [20], was also adopted and modified. The adaptation of the “SP educator” role was closely aligned with the ASPE standards of best practice [7].

The second subgroup compiled professional activities based on the literature reviewed to date, materials from SPE capacity-building activities [24], [25] and members’ day-to-day professional experience. A shared spreadsheet was created to clearly structure and present the complex activities and their subtasks. The detailed breakdown aimed to make the responsibilities of SPEs more understandable and accessible to the intended audience. The professional activities were formulated in line with published guidelines for entrustable professional activities [26], while deliberately omitting the entrustment component in light of the framework’s intended formative application.

The activities overview allowed for the structured integration of newly emerging ideas and aspects. Its accessibility facilitated communication and collaboration, enabling all members to contribute perspectives and content. Both in the subgroups and during online plenary meetings, the structure and content was iteratively expanded and refined based on ongoing members’ feedback.

### 2.5. Interim validation and integration of feedback

After a preliminary draft of the roles and professional activities had been developed, external validation was carried out at a dedicated symposium during a scientific conference on skills and simulated participants in health professions education in Cologne, Germany in 2023 [27]. The draft was systematically reviewed and discussed with approximately 20 SP educators from Germany, Austria and Switzerland, seeking inconsistencies, omissions, and unclear elements. All participants’ perspectives were heard, clarifying questions and feedback helped to eliminate misunderstandings and further specify the draft. Following this activity, the feedback was integrated into the framework during several rounds of internal review within the working group. Subsequently, language and style were refined in editorial meetings. 

### 2.6. Defining SPE competencies

In a subsequent step, the working group identified the competencies and skills necessary to perform the professional activities effectively. It was acknowledged that these competencies are typically distributed across team members within an SP program rather than being embodied in a single individual. 

Recognizing the close relationship between competencies and roles, the group then mapped each professional activity to the previously defined professional roles in a dedicated substep. As a rule of thumb, each activity was assigned to as few roles as reasonably possible, ideally no more than three, unless a broader attribution was explicitly discussed and agreed upon within the group. Thus overly broad or inflated associations were avoided.

### 2.7. Final validation and completion

The final validation of the documents was carried out in several sub-steps:

In a penultimate online plenary meeting, the comprehensive framework with the description of SPEs’ professional roles, professional activities and competencies was reviewed and formally approved as a consensus product accepted by all participants.

Closing the loop on the initial literature review, and in line with the inductive methodology chosen for this project, a systematic comparison was conducted between the emerging framework and existing publications, i.e., the frameworks by Kim et al. [6] and Pritchard et al. [15], both of which are not explicitly reflected in the ASPE SOBP [7], as well as the ASPE SOBP [7] themselves as the widely recognized international standard. This comparison aimed to ensure that all potentially relevant roles, activities, and competencies of SPEs were captured. The comparison yielded 33 differences, prompting the addition of seven professional activities, predominantly at the policy level, as well as thirteen tasks, and eleven task specifications, while the role definitions remained unchanged. Eight items from Kim et al. [6] and the ASPE SOBP [7] diverged from our findings but were not included in the final framework; three were considered too minor to warrant integration, and five were judged not to reflect typical SPE practice in the DACH context.

Although the working group already represented diverse health professions backgrounds, its members were predominantly embedded in medical school contexts. Therefore, as a final validation step, additional feedback was sought from two experts with direct experience in nursing education to assess the framework’s transferability beyond medicine.

## 3. Results

The competency framework for SPEs is structured around three dimensions: the professional roles SPEs assume, the professional activities they carry out, and the competencies required to perform these activities effectively.

The SPE’s professional roles were defined as:

*The central role of SPEs is to apply the SP methodology meaningfully and professionally. They do so within undergraduate, postgraduate, and continuing education in the health professions. They qualify SPs for teaching and assessment assignments in accordance with recognized standards of best SP practice. Moreover, they support and advise educators and institutions in the implementation, quality assurance, and further development of the SP methodology. They thereby contribute to contemporary didactics, a modern educational system, and ultimately to improved healthcare for the general population. SPEs adopt an evidence-informed approach and engage with scholarly discourse. SPE communicate professionally, collaborate in an interprofessional and team-oriented manner, maintain respectful interactions with all individuals involved in the process, and take responsibility for their well-being. They engage critically, constructively, and with a forward-oriented mindset in shaping developments in education and healthcare. Finally, SPE lead and manage SP programs efficiently, effectively, with foresight, and consistently advance both the SP program and the methodology as a whole.*

The eight professional roles identified in this context are those of SP Educator, Scholar, Communicator, Collaborator, Health Advocate, Manager, Professional, and Visionary. The titles and, to some extent, their descriptions, deliberately mirror those of the CanMEDS [20] framework and its German counterpart, NKLM [21], underscoring their shared grounding in health professions education.

Each of these eight roles is further described (cf. attachment 1 ). As an illustrative example, the following text outlines the role of the Visionary. First introduced in the 2021 revision of the NKLM, it highlights a professional’s contribution to curriculum innovation.

*In the visionary’s role, SPE reflect on and expand the boundaries of their own thinking and actions, as well as those of their SP program. They draw inspiration from the environment, technology, and society to further develop the SP method and make themselves available for exchange with others, including those outside the health professions. They anticipate future developments and emerging topics, strategically and proactively align the SP program accordingly, and advocate for corresponding curricular adaptations.*

The SPE’s 39 professional activities are organized into six thematic domains, further subdivided into a total of 132 tasks:

- Building, maintaining, and administering the SP program
- Developing scenarios
- Training SP
- Preparing, delivering and debriefing simulation-based activities
- Assuring Quality
- Developing professional identity and skills

Each professional activity is associated with relevant competencies and mapped to the professional roles assumed by SPEs in the course of performing it. Table 1 (Tab. 1) illustrates this with the example of the professional activity “recruiting new SP”; the full list of professional activities is provided in attachment 2 .

The order of the thematic domains is not meant to imply any differences in their relevance. This framework does not cover simulation technician training, or non-SP-based simulation.

## 4. Discussion

This framework represents a structured proposal for defining the professional roles and activities of SPEs in German-speaking countries, and constitutes the first comprehensive competency-based framework for SPEs worldwide. It responds to recent calls to more closely examine the expertise of SP educators [16], [18]. By mapping roles, activities, and competencies, the framework provides a practice-oriented tool that can help identify gaps or inconsistencies within local SP programs and serve as a roadmap for program development and refinement.

The framework is conceptually aligned with existing SP programs in the DACH region [10], [24] as well as the literature [6], [7], [15] and draws on outcome frameworks from health professions education such as CanMEDS [20] and the German NKLM [21]. The framework is primarily contextualized for DACH countries; however, it may also serve as a source of inspiration for SP programs in other regions. Nevertheless, cultural and systemic differences in the scope of SPE practice must be acknowledged [8]. In many U.S.-based SP programs, for instance, SPs not only portray patient roles and provide feedback to learners but also complete checklists to assess learner performance. This activity entails a distinct set of competencies that are not encompassed within the present framework, as SPs in DACH countries typically do not engage in checklist-based summative assessment as examiners. As the present framework is conceptually grounded in medically oriented reference frameworks (CanMEDS and NKLM), interprofessional collaboration was not its primary organizing perspective. For use in interprofessional education contexts, complementary frameworks such as CIHC [28] or IPEC [29] may provide valuable additional perspectives.

A key strength of the development process was its participatory and consensus-driven approach. Iterative feedback loops and the use of collaborative web-based tools supported transparency, traceability, and completeness. Particular attention was paid to ensuring that all voices were heard and that all participants had the opportunity to contribute meaningfully throughout the process. At the same time, mapping our competency-based framework, developed through an inductive approach, against published frameworks helped ensure that the final product was as comprehensive and complete as possible.

Nevertheless, the chosen methodology also entails limitations. Despite the intentional diversity of the expert group, the framework remains shaped by the expertise, experiences, and institutional contexts of the participating experts, who were predominantly embedded in medical school environments in German-speaking countries. As with all consensus-based approaches, the final framework reflects negotiated agreement and may underrepresent perspectives that were not represented in the working group or the external validation process. Furthermore, the inductive process necessarily involved interpretative decisions regarding the clustering, prioritization, and wording of roles, activities, and competencies. Further validation across other health professions, institutional settings, and cultural contexts is therefore warranted.

It is important to emphasize that this framework was never intended for use in performance evaluation or other summative contexts. The professional activities identified were explicitly not designed as entrustable professional activities (EPAs), and entrustment decisions were not part of the development process. Rather, the framework was conceived as a formative tool to support professional development, facilitate reflection, inform program design, and enhance clarity around the diverse roles of SPEs. Any summative application would go beyond the authors’ original intent.

This framework makes visible the broad and complex scope of SPEs’ work, highlighting the need for qualified professionals who are familiar with SP methodology to take responsibility for program development and delivery. While onboarding into the role is possible, it is a highly specialized domain, and the diverse skill set required to perform these varied tasks effectively and to a high standard should not be underestimated. There is no doubt SP programs increasingly occupy indispensable curricular niches: they enable communicative and interpersonal skills training, underpin OSCEs, and provide forms of human simulation for which no real alternative exists [3]. In many institutions, they have become curricular linchpins, high-stakes, highly visible, and essential for educational quality. If SP programs are entrusted with such central responsibilities, institutions must grant them a clear mandate and the personnel and financial resources required to meet these expectations. Anything less risks a mismatch between curricular demands and the program’s capacity to deliver.

This framework is intentionally focused on those working with, for, and on SPs, and not on broader educational or administrative roles. During development, a clear distinction was drawn between SPEs and other roles such as teaching faculty, simulation technicians, or make-up artists, even if these may overlap in practice. Its competency- and activity-based structure offers institutions practical guidance for recruitment, training, and support of SPEs. In SPE capacity building, training modules could be built around the six activity domains and the professional roles of SPE [25]. Potential uses include job postings, team structuring, program development, and resource allocation. It may also serve as a basis for institutional discussions around professional recognition and appropriate remuneration for SPEs as academic staff working within the “third space”.

The framework can serve as a “blueprint of SPEs’ profession” with a formative, non-prescriptive character. It deliberately does not distinguish between one-time and recurring tasks, or between basic and advanced activities, allowing for flexible adaptation across different stages of program maturity and diverse institutional or cultural contexts. However, this flexibility also requires careful consideration and tailoring to local needs, structures, and resources. Users should also be aware that behind seemingly simple formulations of professional activities often lie complex considerations. For example, defining selection criteria for new SPs involves nuanced decisions around diversity, the ability to give and receive feedback, reliability, and adaptability, all of which require consulting the literature and making informed choices. 

Notably, the framework does not yet explicitly include attitudes or professional values, which are essential to the work of SPEs, though some are implicitly reflected. The identity of an SPE is shaped not only by knowledge and skills, but also by a responsible, empathetic, and respectful stance toward simulated participants and other parties involved. A complementary catalogue of values could meaningfully enrich and complete the framework. Further validation is needed, especially across health professions and cultural settings. Moreover, certain technological developments, such as the integration of AI into simulation, remain unaddressed and could be considered in future iterations.

## 5. Conclusions

We hope that this work provides a solid foundation for both newly established and existing SP programs, offering support in negotiations and helping strengthen the position of SP programs in resource allocation and institutional recognition. As the role of SPEs gains recognition globally, this framework contributes to the professionalization and institutional integration of this vital group.

A complementary set of core values could be developed to capture the ethical and interpersonal dimensions of SPEs’ work. Ongoing discourse, at professional conferences, within institutional committees, and across programs, is encouraged to further refine and adapt the framework. 

## Acknowledgements

The authors would like to express their gratitude towards the participants of their symposium at the iSLS in Cologne for their contributions and thoughtful input, as well as Elvira Pippel (Uppsala County, Sweden) and Claudia Schlegel (Bern, Switzerland) for reviewing the manuscripts final draft from a nursing education perspective.

## Notes

### Authorship

The authors Daniel Bauer and Tanja Hitzblech share the first authorship.

### Authors’ ORCIDs

- Daniel Bauer: [0000-0002-3337-3327]
- Tanja Hitzblech: [0000-0003-0876-0373]
- Peter Eberz: [0000-0003-2763-5682]
- Angelika H. Fritz: [0009-0002-8174-0846]
- Veronika Eva Kühn: [0009-0001-0787-9965]
- Tim Peters: [0009-0008-4165-5608]
- Swetlana Philipp: [0009-0007-5458-3693]
- Beate G. Brem: [0000-0002-0551-9587]

## Competing interests

The authors declare that they have no competing interests. 

## Supplementary Material

Eight professional roles

Complete overview of all professional activities

## Figures and Tables

**Table 1:**
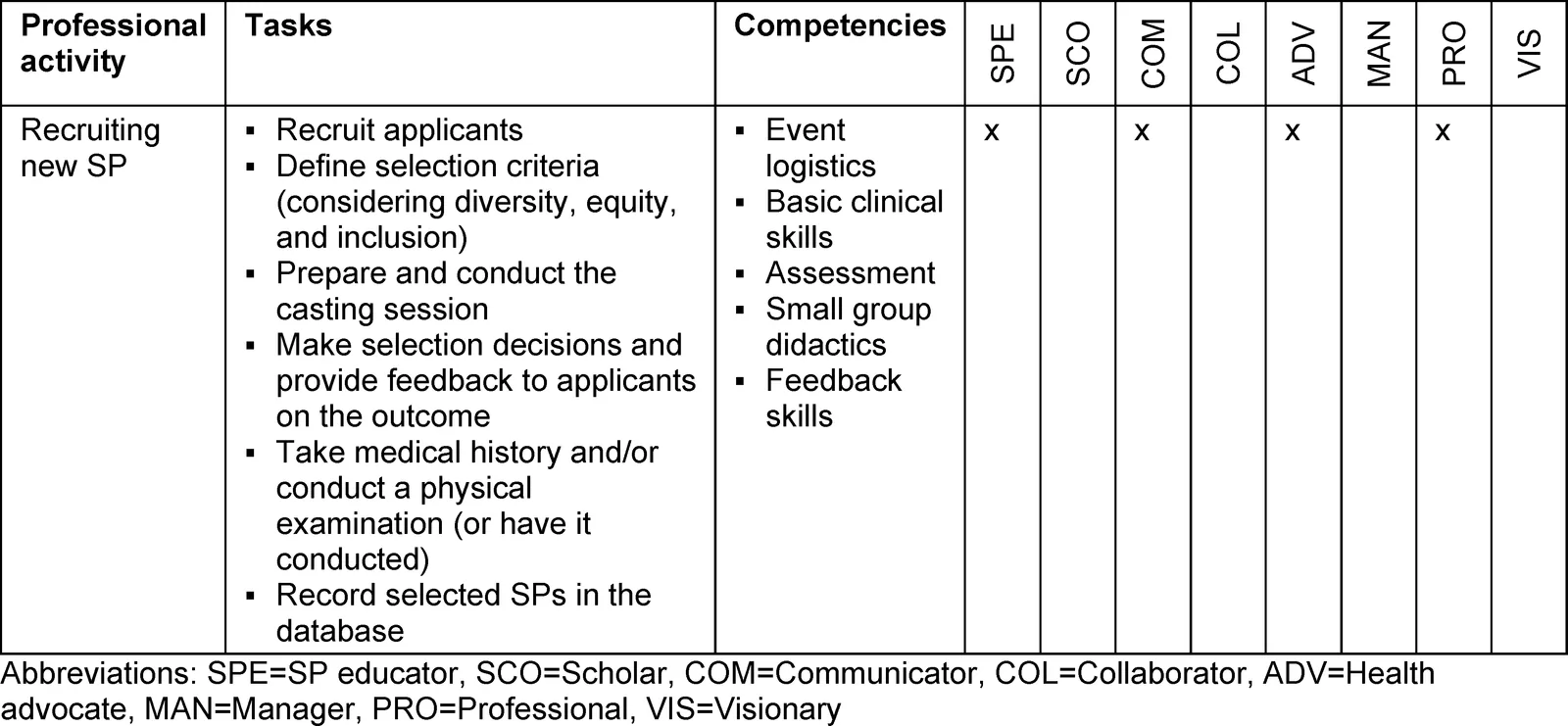
Exemplary professional activity: “Recruiting new SP”, associated tasks, required competencies, and mapping to the most relevant professional roles

## References

[1] Barrows HS (1993). An overview of the uses of standardized patients for teaching and evaluating clinical skills. AAMC. Acad Med.

[2] Barrows HS, Abrahamson S (1964). The programmed patient: A technique for appraising student performance in clinical neurology. J Med Educ.

[3] Bauer D, Lahner FM, Huwendiek S, Schmitz FM, Guttormsen S (2020). An overview of and approach to selecting appropriate patient representations in teaching and summative assessment in medical education. Swiss Med Wkly.

[4] Salden P, Tremp P, Eugster B (2020). Hochschuldidaktik als Teil des Third Space in der Hochschule. Celia Whitchurchs Forschung über Shifting identities and blurring boundaries.

[5] Whitchurch C (2008). Shifting identities and blurring boundaries: The emergence of third space professionals in UK higher education. High Educ Quart.

[6] Kim SH, Lee YH, Yoo HB, Park JH (2012). Job analysis of standardized patient trainer. Korean J Med Educ.

[7] Lewis KL, Bohnert CA, Gammon WL, Hölzer H, Lyman L, Smith C, Thompson TM, Wallace A, Gliva-McConvey G (2017). The association of standardized patient educators (ASPE) standards of best practice (SOBP). Adv Simul (Lond).

[8] Brem BG, Hoelzer H, Blatt B, Ruba E, Miller JL, Smith CM (2023). Advancing professionalization in human simulation: perspectives of SP educators from around the world on the Association of SP Educators Standards of Best Practice. Int J Healthc Sim.

[9] Cleland JA, Abe K, Rethans JJ (2009). The use of simulated patients in medical education: AMEE Guide No 42. Med Teach.

[10] Peters T, Thrien C (2018). Simulationspatienten: Handbuch für die Aus- und Weiterbildung in medizinischen und Gesundheitsberufen.

[11] Peters T, Sommer M, Fritz AH, Kursch A, Thrien C (2019). Minimum standards and development perspectives for the use of simulated patients–a position paper of the committee for simulated patients of the German Association for Medical Education. GMS J Med Educ.

[12] Wind LA, Van Dalen J, Muijtjens AM, Rethans JJ (2004). Assessing simulated patients in an educational setting: the MaSP (Maastricht Assessment of Simulated Patients). Med Educ.

[13] Schnabel KP, Bauer D, Schmitz FM, Hitzblech T, Brem BG (2025). Simulated patients’ role-portrayal in the clinical skills part of the Swiss federal licensing exam is of high quality and improves further over time as measured with the FAIR OSCE instrument. GMS J Med Educ.

[14] Aiello MT, Struijk JS, Szauter K, Nicholas CF, Gliva-McConvey G, Nicholas CF, Clark L (2020). Professional development of the SP educator.

[15] Pritchard SA, Blackstock FC, Keating JL, Nestel D (2017). The pillars of well-constructed simulated patient programs: a qualitative study with experienced educators. Med Teach.

[16] Elcin M, Gliva-McConvey G, Nicholas CF, Clark L (2020). The standardized/simulated patient methodology around the World (Part I).

[17] Cantillon P, Stewart B, Haeck K, Bills J, Ker J, Rethans JJ (2010). Simulated patient programmes in Europe: collegiality or separate development?. Med Teach.

[18] Clark L, Doyle A, Elcin M, McNaughton N, Nicholas C, Owens T, Smith C, Szauter K, Xing K, Nestel D (2025). Call to action: honoring simulated participants and collaborating with simulated participant educators. Simul Healthc.

[19] Huber J, Nepal S, Bauer D, Wessels I, Fischer MR, Kiessling C (2015). Tools and instruments for needs assessment, monitoring and evaluation of health research capacity development activities at the individual and organizational level: a systematic review. Health Res Policy Syst.

[20] Frank JR, Danoff D (2007). The CanMEDS initiative: implementing an outcomes-based framework of physician competencies. Med Teach.

[21] Fischer MR, Bauer D, Mohn K, NKLM-Projektgruppe (2015). Finally finished! National competence based catalogues of learning objectives for undergraduate medical education (NKLM) and dental education (NKLZ) ready for trial. GMS Z Med Ausbild.

[22] Berendonk C, Waldvogel TE, Bonvin R, Schirlo C, Goldhahn J, Frank Z, Nendaz M (2025). Revision of a nationwide competency framework for undergraduate medical education in Switzerland: PROFILES 2. Swiss Med Weekly.

[23] Michaud PA, Jucker-Kupper P, The Profiles Working Group (2016). The" Profiles" document: a modern revision of the objectives of undergraduate medical studies in Switzerland. Swiss Med Wkly.

[24] Strohmer R, Liebke L, Linder U, Kaden JJ (2023). Qualifizierung für ein neues Berufsbild: Training für Simulationspersonen-Trainerinnen und Trainer. ProCare.

[25] Brem B, Bauer D, Hitzblech T, Schnabel K (2024). Ausbildung zum Experten in der Arbeit mit Simulationspersonen (SP) – ein praktisches Beispiel.

[26] Ten Cate O, Taylor DR (2021). The recommended description of an entrustable professional activity: AMEE Guide No. 140. Med Teach.

[27] Bauer D, Brem B, Strohmer R, Peters T (2024). Annäherung an ein Rollen-, Aufgaben-und Kompetenzprofil von SP-Trainer: innen.

[28] Canadian Interprofessional Health Collaborative (2024). CIHC Competency Framework for Advancing Collaboration.

[29] Canadian Interprofessional Health Collaborative (2010). A National Interprofessional Competency Framework.

