# Supplementary material for: Professional roles and activities of simulated patient educators. Development of a multidimensional competency framework
Source: GMS J Med Educ. 2026 Sep 15;43(6):Doc83. doi: 10.3205/zma001877 (PMC13617400; doi:10.3205/zma001877)
Supplement: Supplementary file 1 [file JME-43-83-s-001.pdf]

Attachment 1: Eight professional roles

The central role of SP educators (SPE) is the meaningful and professional application of the SP methodology within the context of undergraduate, postgraduate, and continuing education in the health professions. They qualify SPs for teaching and assessment assignments in accordance with accepted standards of good SP practice, support and advise educators and institutions in the implementation, quality assurance, and further development of the SP method, and thereby contribute to contemporary didactics, a modern educational system, and ultimately to improved healthcare for the population.
They work in a scholarly manner, communicate professionally, collaborate across professions and in teams, maintain respectful interactions with all individuals involved in the process, and take responsibility for their well-being. They engage critically, constructively, and with a forward-looking mindset in shaping developments in education and healthcare, lead and manage SP programs efficiently, effectively, and with foresight, and continuously advance both the SP program and the SP methodology as a whole.

Role of educator

In the role of SP educator in the narrower sense, SPE operationalize SP methodology in their training work with SPs. This includes the conceptual, (theatre-)pedagogical, methodological, and logistical preparation and delivery of training sessions. Within these, they enable SPs to perform specific roles authentically, consistently, and, where applicable, in a standardized manner. They prepare SPs for unexpected situations while considering the specific context and organizational structure of the teaching or assessment activity.

They empower SPs in providing meaningful, individualized feedback or debriefing, considering the setting, process, and learning objectives. SP Educators continuously ensure the quality assurance and ongoing development of their training and implementation concepts.

Role of scholar

In the role of scholar, SP educators work in a scholarly manner through the critical evaluation and application of scientific information and its sources. They maintain and enhance their professional practice through continuous, lifelong learning. They recognize the limits of their own competence and actively seek appropriate ways to expand it. They act as educators for diverse audiences and contribute to the development, dissemination, application, and translation of knowledge and best practices related to SP methodology. Furthermore, they apply the SP method to address research questions from other health- and social-related disciplines in accordance with the highest possible standards.

Role of communicator

In the role of *communicator,* SP educators recognize the central importance of communication skills for collaboration within their own team, with SPs, members of their institution, and third parties. They foster a relationship of trust and responsibility with the SPs. They communicate effectively within interprofessional and interdisciplinary teams, and with representatives of committees, the media, and the public. They model professional and contemporary communication and ensure that these principles are appropriately reflected in teaching and assessment through the SP methodology.

Attachment 1 to Bauer D, Hitzblech T, Eberz P, Fritz AH, Kühn V, Peters T, Philipp S, Strohmer R, Brem BG. *Professional roles and activities of simulated patient educators. Development of a multidimensional competency framework*. GMS J Med Educ. 2026;43(6):Doc83. DOI: 10.3205/zma001877

Role of collaborator

As *collaborators,* SP educators work with SPs, team members, and third parties, often across disciplines and professions, in a spirit of partnership, respect, creativity, and effectiveness. They are skilled in coordinating and integrating collaboration with SPs and various teams to accomplish shared, complex tasks and challenges in teaching and assessment. Ensuring curricular coherence further requires systematic collaboration across institutional and programmatic boundaries.

Role of health advocates

As *health advocates,* SP educators attend to their own well-being as well as that of their team, the SPs, and the learners. They ensure a safe and healthy working and learning environment that supports physical, emotional, and psychological well-being. They apply safe working practices and foster an atmosphere of confidentiality and mutual respect.

Role of manager

In the role of *manager,* SP educators assume responsibility for their teaching and act as active contributors to curriculum design and development. They are familiar with the structures and functions of their (faculty) institutions, organizations, and committees, as well as with the key legal and regulatory frameworks governing education and their own institution. They manage their own professional activities, those of their team, and the SP program efficiently and effectively, applying established principles of quality assurance and quality management.

Role of professional

As *professionals,* SP educators are guided by an ethically grounded and reflective attitude that ensures the meaningful and safe application of the SP method. They maintain confidentiality when handling personal information and treat all individuals with respect, acknowledging the diversity of personal backgrounds and needs. They are always aware of their role as role-model for others and act accordingly.

Role of visionary

In their role as *visionary,* SP experts reflect on and expand the boundaries of their own thinking and actions, as well as those of their SP program. They draw inspiration from the environment, technology, and society to further develop the SP methodology and make themselves available for exchange with third parties, including beyond the health professions. They anticipate future developments and topics and strategically and proactively align the SP program accordingly, while also contributing to curricular adaptations.
