# Supplementary material for: Professional roles and activities of simulated patient educators. Development of a multidimensional competency framework
Source: GMS J Med Educ. 2026 Sep 15;43(6):Doc83. doi: 10.3205/zma001877 (PMC13617400; doi:10.3205/zma001877)
Supplement: Supplementary file 2 [file JME-43-83-s-002.pdf]

Attachment 2: Complete overview of all professional activities

Thematic domain	Professional activity	Tasks included	Knowledge/skills/attitudes competencies	SPE	SCO	COM	COL	ADV	MAN	PRO	VIS
**Building, maintaining, and administering the SP program**	Define and articulate Purpose	* Develop, update, and disseminate mission and vision statements, as well as program objectives	Leadership<br>Educational design<br>Change management	x						x	x
	Create, update, and disseminate informational materials	* Develop materials outlining the role profile and requirements for SPs<br>* Document the general conditions of SP work (rights, duties, boundaries, conflicts of interest)<br>* Record the formal conditions of SP engagement (contractual terms, confidentiality, remuneration, risks)	Legal standards	x					x		
	Recruit SPs	* Recruit applicants<br>* Define selection criteria (considering diversity, equity, and inclusion)<br>* Prepare and conduct the casting session<br>* Make selection decisions and provide feedback to applicants on the outcome<br>* Take medical history and/or conduct a physical examination (or have it conducted)<br>* Record selected SPs in the database	Event logistics<br>basic clinical skills<br>Assessment<br>Small group didactics<br>Feedback skills	x		x		x		x	
	Convey general information and skills	* Clarify the SP’s role as a facilitator in teaching<br>* Explain the differences between various didactic formats and their comparison with stage performance<br>* Teach basic skills in acting and providing feedback<br>* Explain the curriculum and professional culture	Small group didactics<br>Drama-based teaching methods<br>Feedback skills<br>Professional culture	x	x	x					
	Actively maintain relationships with SPs	* Support SPs formally and informally (trust-building measures, availability for concerns)<br>* Provide SP-specific events (e.g., annual meetings) and communication channels (e.g., newsletter)<br>* Welcome SPs to the program and see them off when leaving	Leadership<br>Feedback skills			x			x	x	
	Assign SPs to third parties	* Ensure that third parties uphold the minimum standards of the SP methodology<br>* Clarify legal and administrative details<br>* Forward requests to SPs after weighing interest and in compliance with data protection regulations	Data privacy<br>Legal standards<br>Duty of care				x	x	x	x	
	Manage finances	* Acquire resources, plan the budget, and negotiate fair SP remuneration<br>* Initiate and oversee SP compensation procedures	Accounting<br>Budget planning						x		
	Oversee administrative processes	* Publish data protection regulations (SPs)<br>* Manage contracts (e.g., SPs, team members, third parties)<br>* Clarify tax regulations (e.g., VAT, flat rates)<br>* Archive consent forms and confidentiality agreements	Data privacy<br>Legal standards<br>Tax regulations<br>Duty of care					x	x	x	
	Build and maintain database	* * Collect, update, and, if necessary, delete data regarding contact information, payment details, health information, critical incidents, contracts, assignments, and payment processing	Data processing<br>Data privacy					x	x	x	

Thematic domain	Professional activity	Tasks included	Knowledge/skills/attitudes competencies	SPE	SCO	COM	COL	ADV	MAN	PRO	VIS
**Developing scenarios**	Formulate learning objectives	* Formulate level-appropriate learning objectives, if applicable in collaboration with subject matter experts	Didactics		x		x				
	Develop role scripts	* Write or develop role scripts, if applicable in collaboration with subject matter experts and depending on the format; include elements as needed (e.g., task description, dramaturgy, narrative, props)<br>* Ensure that the role portrayed is plausible and realistic, applying a diversity-sensitive approach<br>* Define requirements for allocating SP<br>* Verify the dramatic and practical feasibility<br>* Determine the degree to which the role is to be standardized or open to interpretation	Didactics drama<br>Pedagogy simulation<br>Methodology	x	x		x				x
	Develop assessment criteria	* Develop feedback criteria and/or checklist items or global rating scales, as appropriate for the format and, if applicable, in collaboration with the team<br>* Integrate available technical solutions (software and hardware)	Didactics<br>Assessment<br>communication models<br>Digital literacy<br>Basic clinical skills	x	x		x				x
	Maintain case database	Organize and archive cases/scenarios and related materials	Data processing						x		
	Produce educational videos	* Provide consultation in developing the script<br>* Produce educational videos, if applicable in collaboration with the team	Media production<br>Didactics	x			x		x		

Thematic domain	Professional activity	Tasks included	Knowledge/skills/attitudes competencies	SPE	SCO	COM	COL	ADV	MAN	PRO	VIS
Training SP	Conduct SP training	*Convey the contextual conditions of the event<br>*Clarify the factual aspects of the role<br>*Develop the role (including appearance, character, expression), proceeding in a diversity-sensitive manner<br>*Define the scope for improvisation<br>*Teach and train the implementation of instructions/directions<br>*Communicate learning objectives / checklist items as needed<br>*Rehearse the use of props, moulages, and makeup<br>*Explain examinations or procedures<br>*Practice the role with different character types of conversation partners<br>*Allow SPs, if applicable, to take on the learners' perspective for role reversal<br>*Have the SP perform the role in a trial run<br>* *Assess whether the SP can perform the role authentically and, if applicable, standardized, and take appropriate actions	Small-group didactics<br>Drama didactics<br>Feedback skills	x					x		
	Follow-up on SP training	*Refer questions to case authors<br>*Adjust case documents<br>* *Provide corresponding feedback/information to SPs	Project management	x		x		x		x	
	Train for Standardization	*Prepare multiple SPs together for the same role<br>*Standardize role performance, if applicable using videos<br>*Identify and reconcile differences in performance<br>* *Train SPs on specific assessment instruments, if needed	Small-group didactics<br>Drama didactics<br>feedback skills	x			x		x		
	Convey feedback techniques and rules	*Facilitate SPs in developing their attitude toward giving feedback<br>* *Prepare feedback rules and concepts	Small-group didactics<br>Feedback skills	x		x					
	Conduct feedback training	*Convey the relevance of specific learning objectives<br>*Teach dialogic feedback, if applicable<br>*Teach and provide appreciative, constructive, and clearly formulated feedback<br>*Train SPs on specific feedback instruments, if needed<br>* *Assess whether the SP feedback meets quality standards and take appropriate actions	Small-group didactics<br>Feedback skills<br>Open-mindedness<br>project management		x	x				x	
	Convey communication models and theories	*Teach communication models to underpin role-portrayal and feedback training<br>* *Teach communication theories to underpin feedback training	Small-group didactics<br>Feedback skills<br>Communication models		x	x				x	
	Conduct SP training	*Convey the contextual conditions of the event<br>*Clarify the factual aspects of the role<br>*Develop the role (including appearance, character, expression), proceeding in a diversity-sensitive manner<br>*Define the scope for improvisation<br>*Teach and train the implementation of instructions/directions<br>*Communicate learning objectives / checklist items as needed<br>*Rehearse the use of props, moulages, and makeup<br>*Explain examinations or procedures<br>*Practice the role with different character types of conversation partners<br>*Allow SPs, if applicable, to take on the learners' perspective for role reversal<br>*Have the SP perform the role in a trial run<br>* *Assess whether the SP can perform the role authentically and, if applicable, standardized, and take appropriate actions	Small-group didactics<br>Drama didactics<br>Feedback skills		x	x				x	

Thematic domain	Professional activity	Tasks included	Knowledge/skills/attitudes competencies	SPE	SCO	COM	COL	ADV	MAN	PRO	VIS
**Preparing, delivering and debriefing simulation-based activities**	Administer the event	* Coordinate session frameworks and workflows with relevant parties<br>* Create, populate, and process datasets for event administration<br>* Book rooms, if applicable<br>* Plan personnel resources, if applicable	Data processing<br>Ressource management<br>Event logistics			x	x		x		
	Plan SP assignments	* Plan SP assignments to minimize travel and transitions<br>* Make assignments attractive for SPs (effort vs. remuneration)<br>* Ensure safe role allocation for SPs (e.g., SP health, expected emotional/physical strain, age)<br>* Ensure safe conditions during assignments (duration, breaks)<br>* Request SPs for specific roles<br>* Send assignment confirmations (including dates and locations)<br>* Plan for absences and last-minute replacements	Data processing<br>Ressource management<br>Duty of care<br>Event logistics	x				x	x		
	Plan and order materials	* Order equipment (simulators, consumables)<br>* Plan, produce, or order moulages	Material and inventory logistics<br>Basic knowledge of make-up artistry/ prosthetics	x					x		
	Prepare the event	* Provide teaching and learning materials<br>* Set up rooms<br>* Prepare materials<br>* Convey content-related information to SPs and, if applicable, to instructors and subject matter experts<br>* Oversee or perform SP makeup and prepare/check models, simulators, and props<br>* Facilitate a final dry-run of the scenario at the beginning of the activity, if needed<br>* Ensure a safe working environment<br>* Serve as a point of contact for SPs	Facility management<br>Basic knowledge of make-up artistry/ prosthetics<br>Event logistics	x			x	x	x		
	Manage problems during implementation	* Manage last-minute SP absences (reassign, train replacements)<br>* Handle defects in (hybrid) models or simulators<br>* Manage conflicts<br>* Serve as a point of contact for SPs	Conflict management<br>Leadership event logistics<br>Basic knowledge of simulators	x		x			x	x	
	Conduct debriefing	* Conduct the session debriefing<br>* Support SPs in de-roling, and, if needed, help manage and process potentially stressful emotions	Feedback skills<br>Coaching/supervision	x		x		x		x	

Thematic domain	Professional activity	Tasks included	Knowledge/skills/attitudes competencies	SPE	SCO	COM	COL	ADV	MAN	PRO	VIS
**Assuring quality**	Monitor quality	* Monitor, assess, and document SP performance in role portrayal and feedback using methods such as observation, evaluation, and video analysis<br>* Solicit feedback on one's own work as an SP expert and on the SP program through 360° feedback mechanisms	Feedback skills<br>Open-mindedness<br>Reflective competence Quality management	x	x			x			x
	Further develop quality	* Analyze quality control data to identify errors and areas for improvement<br>* Adapt or redesign work processes and products (e.g., scripts, methodologies, events) based on findings<br>* Facilitate continuing education and professional development for SPs	Open-mindedness<br>Change Management Quality Management	x		x				x	
	Conduct faculty development	* Conduct faculty development on the meaningful application of the SP methodology ("SP license")<br>* Conduct examiner trainings<br>* Supervise and provide feedback to instructors and examiners<br>* Evaluate and/or seek certification for the SP program	Small-group didactics<br>Feedback skills Assessment evaluation	x						x	x
	Conduct curriculum development	* Identify and address curricular gaps where the SP methodology could be effectively applied<br>* Identify existing courses or sessions that could benefit didactically from SP methodology and support their modernization	Curriculum planning	x	x	x				x	
	Further develop the SP methodology	* Conduct and publish scientific studies<br>* Publish project reports on innovative applications of the SP methodology	Research literacy<br>Project management	x	x						x
	Participate in continuing education and training	* Participate in internal and external trainings, workshops, and professional conferences<br>* Attend practical workshops to enhance skills	Research literacy	x	x						x
	Monitor quality	* Monitor, assess, and document SP performance in role portrayal and feedback using methods such as observation, evaluation, and video analysis<br>* Solicit feedback on one's own work as an SP expert and on the SP program through 360° feedback mechanisms	Feedback skills<br>Open-mindedness<br>Reflective competence Quality management	x	x						x
**Developing professional identity and skills**	Self-development and self-care	* Continuously develop professional skills and competencies as an SP Educator<br>* Manage personal well-being, time, and workload effectively	Reflective competence<br>Introspection		x			x	x		
	Team management and career development	* Ensure adequate personnel resources are available to support the SP program's activities<br>* Ensure that tasks can be completed efficiently and effectively within the team<br>* Engage with a career mentor and provide mentorship to others	Mentoring<br>Project management<br>Leadership			x	x		x		
	Maintain collaborations	* Engage in networking and collaborative activities within committees and working groups (community of practice)<br>* Foster interprofessional and interdisciplinary collaboration at faculty, national, and international levels	Networking	x		x	x				
	Conduct research projects	* Secure and allocate resources<br>* Conceptualize, execute, and publish projects and studies, individually or collaboratively	Research literacy<br>Project management	x	x		x				x
	Design and implement public relations activities	* Develop targeted content and utilize appropriate communication channels or events to represent and promote the SP program externally	Media literacy<br>Science communication	x		x	x				

Attachment 2 to Bauer D, Hitzblech T, Eberz P, Fritz AH, Kühn V, Peters T, Philipp S, Strohmer R, Brem BG. *Professional roles and activities of simulated patient educators. Development of a multidimensional competency framework*. GMS J Med Educ. 2026;43(6):Doc83. DOI: 10.3205/zma001877
